# Development of methods for data quantitation of spiked salmon host cell DNA in protamine sulfate by qPCR

**DOI:** 10.1016/j.dib.2018.09.135

**Published:** 2018-10-04

**Authors:** Cynthia Sommers, Barry Rosenzweig, Lida Oum, Karol Thompson, David A. Keire

**Affiliations:** a Food and Drug Administration, CDER, Division of Pharmaceutical Analysis, St Louis, MO 63110, USA; b Food and Drug Administration, CDER, Division of Applied Regulatory Science, Silver Spring, MD 20993, USA; c Food and Drug Administration, CDER, Division of Product Science, Silver Spring, MD 20993, USA

**Keywords:** Protamine sulfate, Real-time polymerase chain reaction, Residual host cell DNA, TaqMan, DNA reference standard

## Abstract

Protamine sulfate (PS) is an approximately 4 kDa cationic polypeptide derived from *chum* salmon used to reverse heparin-induced anticoagulation in patients. Because the presence of residual host cell salmon DNA (resDNA) in PS drug product can pose safety concerns, processing steps during PS manufacturing are designed to target the reduction of these impurities. However, given protamine׳s positively charged structure, isolating and measuring negatively charged residual DNA is challenging. Suitable resDNA methods for PS require the generation of host DNA reference materials, efficient DNA extraction procedures and assay sensitivity and accuracy as high as possible. Here, optimization data are shown for the extraction of DNA present in PS drug products and for the generation of reference standard from protease-digested research grade chum salmon DNA. The lower limit of quantitation (LLOQ) for the reference standard determined from protease-digested DNA (0.0025–156.25 pg/μL) was 0.0025 pg/μL. The extraction procedure LLOQ, determined from DNA (0.01–1.25 pg/μL) spiked into PS samples, was 5 pg DNA per mg PS. The data supporting the LLOQs were evaluated using acceptance criteria of 70–130% recovery with % correlation coefficient (CV) ≤ 25% for DNA concentrations and curve metrics (slopes, *R*^2^ and y-intercepts) within 2SD of the mean. The data presented here complement a broader study (Sommers et al., 2018) [1] and are particularly useful for the development of resDNA methods for challenging drug products.

**Specifications table**

**Table:** 

Subject area	*Drug Product Safety*
More specific subject area	*Residual host cell DNA*
Type of data	*Tables and Figures*
How data was acquired	*Real-time quantitative polymerase chain reaction (qPCR) assay for a multicopy gene (5S ribosomal DNA) using custom-designed primers and TaqMan probes*
Data format	*Analyzed*
Experimental factors	*Saline, PS or TE buffer samples were spiked with protease-digested salmon DNA (0.01–1.25 pg/μL).*
Experimental features	*DNA was extracted using modifications to the RecoverAll® protocol. Percent recovery was calculated from salmon DNA standard curves (0.0025–156.25 pg/μL) by qPCR.*
Data source location	*FDA Division of Applied Regulatory Science, White Oak, Maryland*
Data accessibility	*Data are available in this article and in Data in Brief Excel Data.xls*

**Value of the data**

• The data show an improved elution procedure which significantly increased the overall extracted DNA accuracy (% recovery) from an average of 42.8% recovery (100 uL) to 90.9% recovery (200 uL).
• The data show a protease digestion method for generating a consistent and reliable DNA reference standard and spiking material that is suitable for measuring resDNA from PS samples, reproducibly.
• The data show intra-assay and inter-assay precision curve metrics and the associated acceptance criteria status for DNA recovered from saline, PS and TE buffer solutions from 8 to 12 independent experiments.
• The data provide a standard, using defined acceptance criteria, of how to determine a procedure LLOQ for resDNA methods using DNA recovery curves from DNA spiked drug product.

## 1 Data

### 1.1 Optimizing DNA extraction

**Fig. 1:**
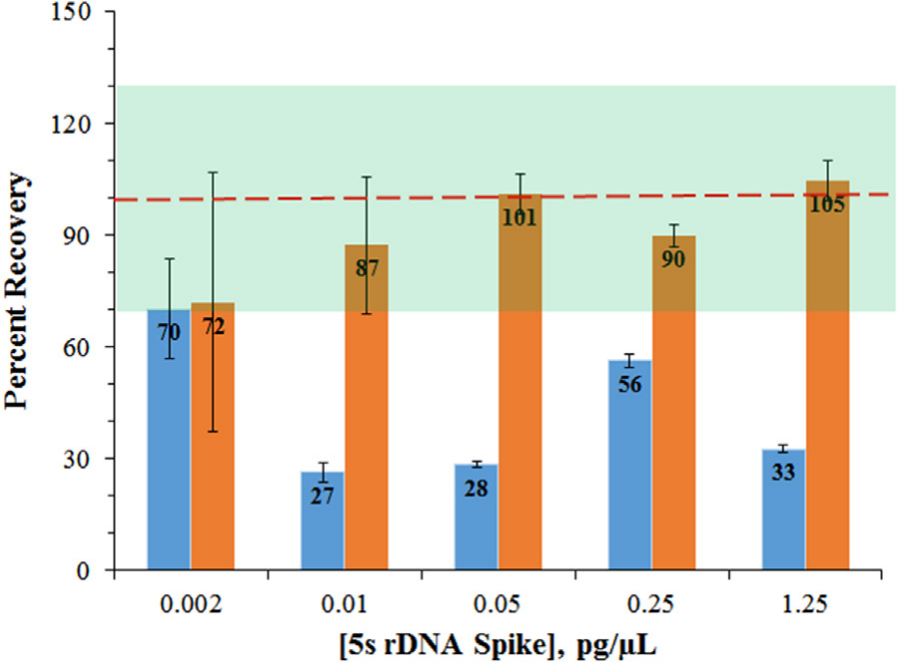
Optimization of DNA elution volume. Plot of average ± SD percent recoveries of salmon DNA by assaying 5s salmon rDNA in spiked TE samples following a single 100 μL (blue bars) or two 100 μL (red bars) column elution steps. Each bar represents the mean intra-assay % recovery of three technical replicates. The RecoverAll® DNA extraction kit was utilized with TE as elution buffer. Percent recovery of DNA from spiked samples (0.002–1.25 pg/μL) was measured by qPCR analysis. Curve equations (slope and y-intercepts) and sample recovery values were extrapolated from a 7-point salmon sperm DNA standard curve, run in triplicate dilutions, ranging from 0.01 to 156 pg/μL. DNA quantity was calculated using the following equation: 10^ (*Ct* (threshold cycle)-y intercept)/slope. Percent recovery, *y* axis and denoted within the bars, was calculated from background corrected *Y* values (DNA quantity) divided by the amount of DNA input * 100. Green highlighted area denotes values within a 70–130% recovery range.

We tested the RecoverAll® kit, the PrepSEQ® kit and Arg-C digestion methods and found the RecoverAll® kit most amenable for optimization given its ease, throughput and high DNA recoveries from saline (PS excipient) solutions [1]. The RecoverAll® kit was further optimized by evaluating 1) digestion times, 2) elution volumes with TE buffer, 3) RNase requirement and 4) column drying conditions. Salmon DNA (0.002–1.25 pg/μL) was spiked into TE buffer samples to evaluate the role of elution volume on % recovery. As shown in Fig. 1, doubling the elution volume from a single 100 μL aliquot to two 100 μL aliquots significantly increased the overall % recovery or accuracy from an average of 42.8% (range 27–70%; 100 μL) to 90.9% recovery (range 72–105%; 2 × 100 μL;). The DNA recovery curve, following two 100 μL elutions also showed greater linearity (*R*^2^ = 0.999) and alignment with DNA concentrations assuming 100% recovery compared with the linearity (*R*^2^ = 0.971) and curve alignment following a single 100 μL elution (Appendix A).

### 1.2 Optimizing DNA concentrations

**Fig. 2:**
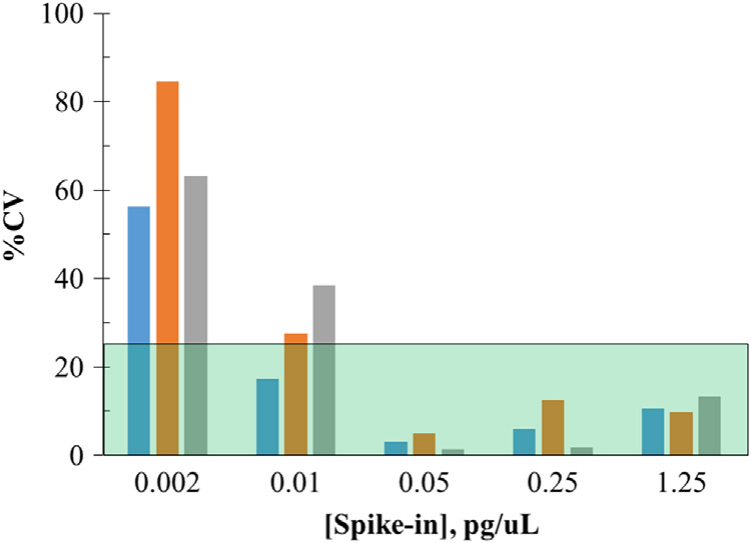
Optimization of DNA Spike-in concentrations. Plot of intra-assay % CV values from % recoveries of salmon DNA (0.002–1.25 pg/μL) spiked into saline samples. The data are from three independent experiments (blue, orange and grey bars). Each bar represents the mean of intra-assay % CVs of three to five technical replicates. The RecoverAll® DNA extraction kit was utilized with TE as elution buffer. Percent recovery of DNA from spiked samples (0.002–1.25 pg/μL) was measured by qPCR analysis using a 7-point salmon sperm DNA standard curve, run in triplicate dilutions, ranging from 0.01 to 156 pg/μL. DNA quantity was calculated using the following equation: 10^ (*Ct* (threshold cycle)-y intercept)/slope. Percent recovery was calculated from background corrected *Y* values (DNA quantity) divided by the amount of DNA input * 100. Green highlighted area denotes % CV values within 25%.

Procedure LLOQs and validation of resDNA methods require spiking host cell DNA into drug product. To select suitable DNA spiking concentrations, optimization tests were performed to evaluate the accuracy (% DNA recovery) and precision (% CV) of salmon DNA (0.002–1.25 pg/μL) spiked into saline samples. Intra-assay (Fig. 2) and inter-assay (Table 1) data show significant increases in % CV values with decreasing DNA concentrations, indicating a loss of assay precision, particularly at the 0.002 pg/μL DNA concentration. There was also an increase in % recoveries noted at the lowest DNA concentrations (Table 1). Nevertheless, the inter-assay % recoveries and % CV values from DNA (0.01–1.25 pg/μL; excluding 0.002 pg/μL) spiked samples were within 70–130% recovery with % CV ≤ 25%, supporting the use of these concentrations for further optimization and LLOQ studies.

**Table 1:** Inter-assay precision metrics for indicated digested DNA spike-in concentrations from saline samples.

**[Nominal Spike DNA], pg/µL**	**[AVG Measured DNA], pg/µL**	**[AVG Measured DNA] SD**	**[AVG Measured DNA] %CV**	**N size**	**% DNA Recovery**	**DNA Recovery %CV**
0.002	0.003	0.001	39.1	3	130.6	39.1
0.010	0.012	0.003	21.3	3	118.5	21.3
0.050	0.053	0.008	15.0	11	105.2	15.0
0.250	0.241	0.038	15.7	11	96.4	15.7
1.250	1.198	0.143	11.9	11	95.9	11.9

### 1.3 Generation of DNA reference standard

**Fig. 3:**
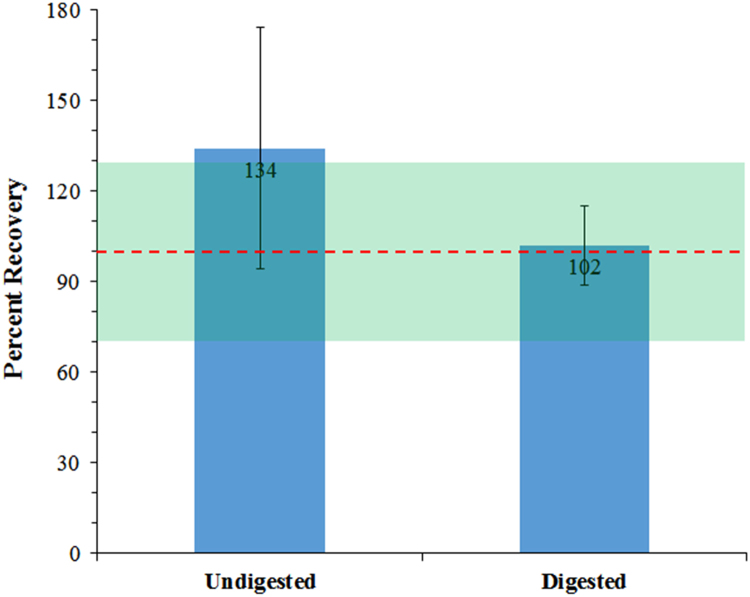
Generation of Salmon DNA Reference standard. Plot of percent recoveries of salmon DNA (0.01–1.25 pg/μL) spiked into TE buffer using untreated DNA (left bar) or protease-digested DNA (right bar) as reference standard. Percent recovery data represent mean ± SD from five to fifteen DNA recovery concentrations. Digested DNA was prepared by incubating 10 μL of salmon DNA (0.01–1.25 pg/μL) plus a cocktail containing RecoverAll® digestion buffer and protease for 60 min at 50 °C, 15 min at 80 °C and purification using the RecoverAll® protocol. Percent recovery of DNA was measured by qPCR. Curve equations (slope and y-intercepts) and sample recovery values were extrapolated from a 7-point salmon sperm DNA standard curve, run in triplicate dilutions, ranging from 0.01 to 156 pg/μL. DNA quantity was calculated using the following equation: 10^ (*Ct* (threshold cycle)-y intercept)/slope. Percent recovery, *y* axis and denoted within the bars, was calculated from background corrected *Y* values (DNA quantity) divided by the amount of DNA input * 100. Green highlighted area denotes values within a 70–130% recovery range.

DNA recovery values were greater than 100% in several of our DNA spiked saline and TE buffer samples. To further understand this observation, the DNA template was digested. Fig. 3 shows the comparison of spike-in control DNA recoveries from TE buffer using undigested DNA (134% ± 35%) compared with protease-digested DNA (102% ± 15%), showing that DNA values were overestimated and more variable using untreated, randomly sheared DNA. The DNA extraction curves from protease-digested DNA (0.01–1.25 pg/μL) spiked into saline samples were also very comparable across 8 independent experiments run over the course of six months using the digested DNA reference standard for qPCR analysis (Table 2A). These data show a method for generating a consistent and reliable DNA reference standard and spiking material that is suitable for measuring resDNA from PS samples, reproducibly.

**Table 2A:** Inter-assay precision metrics for 8 independent DNA spike in titration curves^a^ from protamine sulfate vehicle (saline).

**Run #**	**Slope**	**y-int**	***R***^**2**^
1	0.989	−0.023	1.000
2	0.947	−0.063	0.999
3	0.955	−0.040	1.000
4	0.947	−0.048	1.000
5	1.014	−0.068	1.000
6	0.924	−0.037	0.999
7	1.023	−0.089	0.999
8	0.965	−0.066	1.000
AVG	0.970	−0.054	0.999
SD	0.035	0.021	0.001
2SD	0.070	0.042	0.001
AVG + 2SD	1.040	−0.012	1.001
AVG − 2SD	0.901	−0.096	0.998

a DNA spike-in concentration range 0.05–1.25 pg/μL.

### 1.4 Establishing DNA extraction curve LLOQ

We adapted well established criteria for sensitivity [2] and % recovery [3] for determining the procedure LLOQ [1]. The first set of criteria require DNA spike-recovery curve parameters to have *R*^2^ values ≥ 0.99 (fitting a 1st order polynomial) and curve slopes and y-intercepts within 2SD limits. The second set of criteria require recovery of DNA spike-in concentrations to be within a range of 70–130% with ≤ 25% CVs. Table 2A, Table 2B show that inter-assay precision curve metrics for DNA recovered from saline (PS vehicle) solutions (2A) and from PS solutions (2B) meet the first set of criteria. Table 2C, Table 2D show the measured DNA concentrations and corresponding % recoveries for DNA recovered from saline (2C) and from PS (2D) across 8 experiments. The range of DNA recovered from spiked samples across the 8 experiments was 80–121% and 57–109% from saline and PS extractions, respectively, all within 2SD of the mean. The averaged % recoveries for DNA (0.05–1.25 pg/μL; range 90–101%) spiked saline and DNA (0.01–1.25 pg/μL; range 84–77%) spiked PS were within 70–130% recovery with ≤ 25% CV (highlighted rows in Table 2C, Table 2D). Collectively, the curve metrics and averaged inter-assay data would support a procedure LLOQ of 0.01 pg/μL. However, three of eight individual run % recovery values for the 0.01 pg/mL spiked PS samples were <70% and had intra-assay CV values >25%. Since all the data for 0.05 pg/μL (highlighted in green) met acceptance criteria, this DNA concentration was chosen as the procedure LLOQ (5 pg DNA per mg PS).

**Table 2B:** Inter-assay precision metrics for 8 independent DNA spike in titration curves^a^ from protamine sulfate solutions.

**Run #**	**Slope**	**y-int**	***R***^**2**^
1	0.972	−0.145	0.999
2	0.948	−0.072	1.000
3	0.939	−0.157	0.998
4	0.985	−0.102	0.999
5	1.026	−0.132	0.994
6	0.966	−0.075	0.999
7	0.985	−0.187	0.998
8	1.067	−0.054	1.000
AVG	0.986	−0.116	0.998
SD	0.042	0.047	0.002
2SD	0.084	0.094	0.004
AVG + 2SD	1.070	−0.022	1.002
AVG − 2SD	0.902	−0.210	0.995

a DNA spike-in concentration range 0.01–1.25 pg/μL.

**Table 2C:** Inter-assay precision metrics for indicated spike-in nominal concentrations from protamine sulfate vehicle (saline).^a^

a From 8 independent experiments for 1.25-0.05 pg/μL and one experiment for 0.01 pg/μL.

**Table 2D:** Inter-assay precision metrics for indicated spike-in nominal concentrations from protamine sulfate solutions.^a^

a From 8 independent experiments using 3 lots of PS.

## 2 Experimental design, materials and methods

### 2.1 Reagents and samples

Protamine Sulfate lots, supplied by Fresenius Kabi USA, were purchased through Bradley Drugs (Bethesda, MD). A conserved region of the multicopy gene for 5S ribosomal RNA (rRNA) from *Salmo salar* sequences (GenBank Accession No. S73107.1) [4] was selected as a target for quantification by qPCR. TaqMan primers and probes were designed against this target using Primer Express 3.0 Software (Applied Biosystems, Foster City CA). TaqMan® Universal Master Mix, DNA Template (Ultrapure salmon Sperm DNA Cat #15632011) and the RecoverAll® DNA extraction kits were supplied through ThermoFisher Scientific (Dallas, TX). Of note, reagent grade chum salmon sperm DNA was used as the DNA reference material given the lack of true DNA reference standards for chum salmon.

### 2.2 DNA extraction

For generation of DNA RM (referred to as DNA), 100 μL of commercially available salmon sperm DNA was protease digested (using protease supplied in the RecoverAll® kit) for 60 min at 50°C, followed by 15 min at 80 °C to heat kill the protease. The digested DNA (8.76 μg/μL), was diluted to a stock concentration of 10 ng/μL in water and stored at 4°C.

To assess percent recovery of DNA through the PS sample extraction process, digested DNA was spiked into samples which were then protease-digested again at 50 °C for 60 min and further processed as per RecoverAll® kit instructions. Ten μL of 10X DNA positive spike-in controls ranging from 0.1 to 12.5 pg/μL (diluted in TE buffer) were added to either 100 μL of PS (1 mg, 10 mg/mL solution), 100 μL of PS diluent (sodium chloride (NaCl), 9 mg/mL, referred to as saline) or 100 μL of Tris EDTA (TE) extraction buffer. Tris EDTA buffer (10 μL) was used instead of DNA for a no spike control for each diluent. Following the digestion step, 790 μL of “Isolation Additive/Ethanol” buffer from the RecoverAll® kit was added to each sample and thoroughly mixed through inversion. Each sample was filtered and washed sequentially with ethanol solutions. The following changes were made to the RecoverAll® protocol: 1) 60 min protease digestion, 2) no RNase treatment, 3) 5 min spin vs 30 s to dry columns after last wash and after final elution and 4) elution with two 100 μL volumes of TE buffer vs once with 60 μL of “Elution” buffer. The averaged recovered volume, 197 μL, was used as a volume correction factor for extrapolation of unknowns in the data analysis.

### 2.3 qPCR method

Nine-point DNA standard curves, ranging in concentration from 0.0025 to 156.25 pg/μL, were generated from serial dilution of protease digested salmon sperm DNA in TE buffer. A 20x TaqMan assay stock was prepared containing 6 μM primers and 5 μM probe. A TaqMan MasterMix (MM) was prepared containing 11 μL of 2x TaqMan® MasterMix II (No Ung) and 1 μL of 20x TaqMan assay stock per assay well. Twelve μL of MM was added to each well, followed by 10 μL of standard, digested sample (with or without DNA spike) or TE buffer (no template control). Twenty μL of this reaction was transferred for qPCR analysis. The plate was read using an Applied Biosystems 7900HT PCR instrument with FAM detection and the following thermal cycling conditions: Step 1: Polymerase activation for 10 min at 95°C, Step 2 DNA denaturation at 95°C for 15 s and Step 3 annealing and extension at 60°C for 60 s for 40 cycles.

### 2.4 qPCR calculations

Standard curve plots were generated from instrument export data; log input [DNA] values plotted on the *x*-axis versus *Ct* values plotted on the *y*-axis. Standard curve statistics including slope, y intercept, *R*^2^ and % efficiency were determined using Excel 2010. DNA values were extrapolated from the curve *y* = *mx* + *b*, where *x* = log input [DNA Reference Standard], *y* = *Ct* value, *b* = y-intercept, and *m* = slope. Therefore, *x* = 10^ ((*y*-*b*)/*m*), or “Extrapolated Unknown” = 10^ (*Ct* – y-intercept)/slope. A no template control (NTC, 10 μL TE buffer) was run in each experiment to control for PCR components alone. The number of experiments or replicates used to determine the data presented are provided in each figure legend or table.

### 2.5 Curve statistics

For effective evaluation of Ct inter-run variance (intermediate precision); inter-run slopes, *R*^2^ and y-intercepts were compared using the criteria of 2SD for the set of curves. The DNA standard curve had to meet requirements of curve linearity (1st order polynomial fit and *R*^2^ > 99%) in addition to meeting slope accuracy and y-intercept variance within 2SD for all individual curves containing the LLOQ concentration. The LLOQ for the DNA standard curve (0.0025–156.25 pg/μL) was determined as the lowest concentration of the curve meeting the following parameters: *R*^2^ > 0.99%, efficiencies ≥ 90%, 1st order polynomial fit and SDs < 0.5. The procedure LLOQ was determined from DNA spike-recovery curves (0.01–1.25 pg/μL) meeting the criteria above in addition to having recovery of DNA from spiked PS samples within a range of 70–130% and CVs ≤ 25%.
